# Designing artificial 2D crystals with site and size controlled quantum dots

**DOI:** 10.1038/s41598-017-08776-3

**Published:** 2017-08-30

**Authors:** Xuejun Xie, Jiahao Kang, Wei Cao, Jae Hwan Chu, Yongji Gong, Pulickel M. Ajayan, Kaustav Banerjee

**Affiliations:** 1 Department of Electrical and Computer Engineering, University of California, Santa Barbara, California 93106 USA; 2 Department of Materials Science and Nanoengineering, Rice University, Houston, Texas 77005 USA

## Abstract

Ordered arrays of quantum dots in two-dimensional (2D) materials would make promising optical materials, but their assembly could prove challenging. Here we demonstrate a scalable, site and size controlled fabrication of quantum dots in monolayer molybdenum disulfide (MoS_2_), and quantum dot arrays with nanometer-scale spatial density by focused electron beam irradiation induced local 2H to 1T phase change in MoS_2_. By designing the quantum dots in a 2D superlattice, we show that new energy bands form where the new band gap can be controlled by the size and pitch of the quantum dots in the superlattice. The band gap can be tuned from 1.81 eV to 1.42 eV without loss of its photoluminescence performance, which provides new directions for fabricating lasers with designed wavelengths. Our work constitutes a photoresist-free, top-down method to create large-area quantum dot arrays with nanometer-scale spatial density that allow the quantum dots to interfere with each other and create artificial crystals. This technique opens up new pathways for fabricating light emitting devices with 2D materials at desired wavelengths. This demonstration can also enable the assembly of large scale quantum information systems and open up new avenues for the design of artificial 2D materials.

## Introduction

The evolution of information technology is approaching the quantum era. Quantum dots or artificial atoms are promising medium for quantum information teleportation and processing^1–3^, and they also represent the state-of-art of human capability to manipulate matter^4^. Recently, *native defects* in 2D layered semiconductor materials have been shown to be a promising single photon source for quantum optics and as potential *qubits* for quantum computing^5–9^. However, for practical applications, it’s critical to control the size and site of the quantum dots to precisely regulate the energy level and couple with photonic crystal cavities to integrate with large scale photonic systems^10–12^. On the other hand, the ultra-thin-body nature of 2D materials allows extraordinary performance advantages for both electronic and photonic devices^13, 14^. However, it’s challenging for 2D materials to possess the desired direct bandgap while preserving strong photoluminescence^15–17^. Quantum dot superlattices have been widely investigated for creating materials with tunable bandgaps^18, 19^. However, they were all made by bottom-up methods that make it inherently hard to design the lattice structure as desired, and their performance get smeared by energetic and positional disorders^20^. Phase transition of MoS_2_ from semiconducting 2H phase to metallic 1T phase by electron beam irradiation has been demonstrated in refs 21 and 22. However, the method in ref. 21 requires temperatures above 400 °C in scanning transmission electron microscope (STEM), thus not suitable for practical and scalable applications. Note that, the work in ref. 23 also irradiated the MoS_2_ in STEM. However, their irradiation dose is too high, which only leads to sulfur vacancies. The work in ref. 22 is carried out on multilayer MoS_2_, and can only achieve micro-scale heterostructures, and thereby the ultimate precision potential of monolayer 2D material is not uncovered. Moreover, none of these previous works created the quantum dots so close to each other in 2D materials that is necessary to promote quantum dot to quantum dot interactions. Here we report the first successful demonstration of a top-down method for creating large area quantum dot superlattice on monolayer MoS_2_ at room temperature by focused electron beam irradiation with sub-nanometer precision in large scale (30 $\mu$m × 30 $\mu$m). We demonstrate that by controlling the size and the lattice spacing of the 2D quantum dot superlattice, we can tune the bandgap of the monolayer MoS_2_ from 1.81 to 1.42 eV. Our work constitutes a photoresist-free top-down method for designing artificial 2D crystals, paves the way for creating large-scale quantum information systems, as well as opens up new pathways for fabricating light emitting devices with 2D materials at desired wavelengths.

## Methods

Monolayer MoS_2_ is synthesized by chemical vapor deposition (CVD) by using sulfur (S) and molybdenum oxide (MoO_3_) powder as the precursors. A Si/SiO_2_ wafer with 285 nm silicon dioxide (SiO_2_) grown on silicon is placed above the MoO_3_ powder with face down as the growth substrate. The boat with MoO_3_ powder and Si/SiO_2_ wafer is then placed in a fused quartz tube, which is located at the center of the CVD furnace. The furnace temperature is raised to 750 °C for 15 minutes and then held at this temperature for 20 mins. S powder is located at the upstream region of the furnace at 200 °C. During the entire process, 50 sccm argon is used as the carrier gas and the growth is allowed under atmospheric pressure. Then we perform the electron beam irradiation at room temperature on the monolayer MoS_2_ with FEI XL-30 SIRION Scanning Electron Microscope (SEM) with Nanometer Pattern Generation System (NPGS) to manipulate the electron beam. The electron beam voltage is 30 kV, and the beam current is 580 pA on spot size 4. The electron beam is made to spot the monolayer MoS_2_ surface point-by-point with designed lattice spacing and point dose (in units of fC). The focused electron spot size is about 2 nm as discussed in the Supplementary Information S1. The focus quality of the electron beam is crucial for the 1T phase transition.

## Results

**Figure 1:**
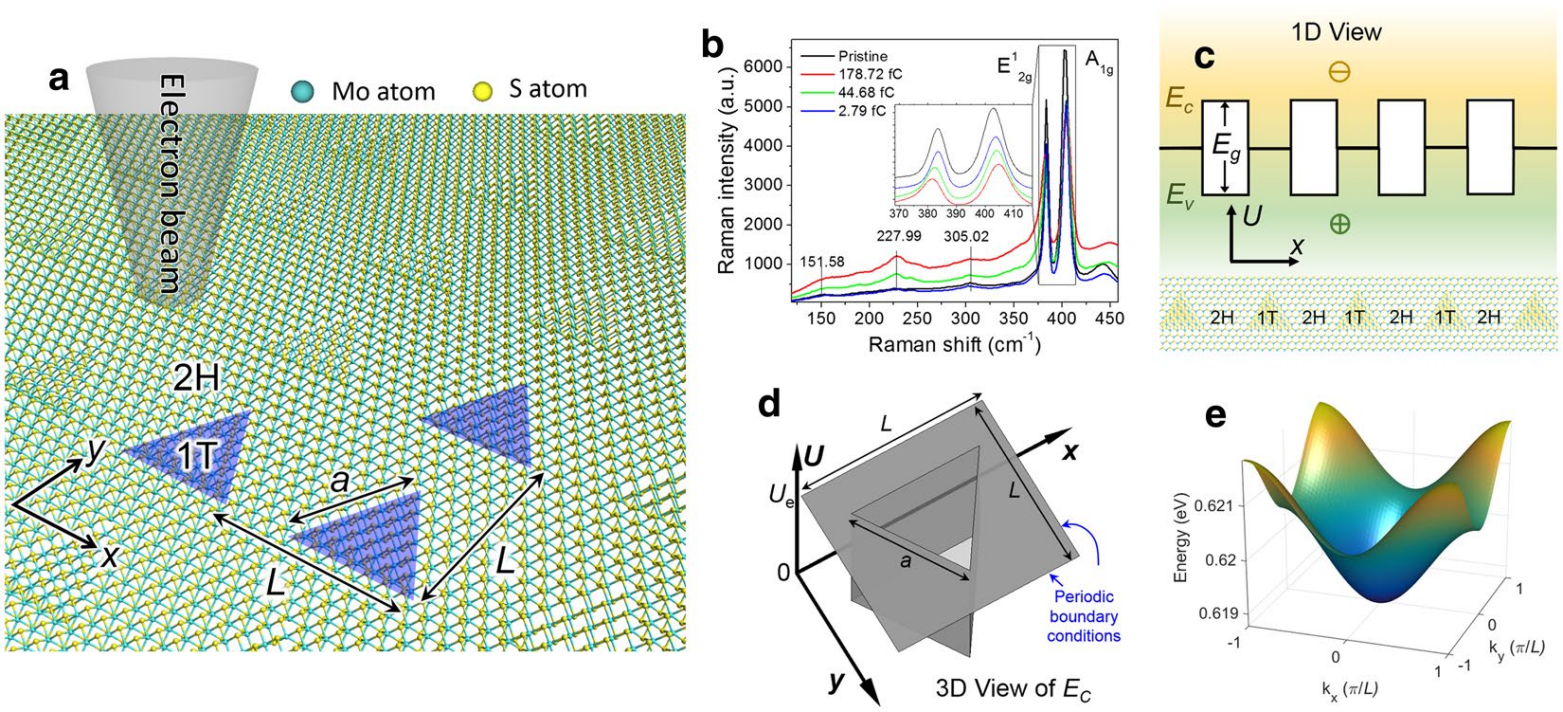
1T phase quantum dot superlattice created on 2H phase monolayer MoS_2_ at room temperature. (**a**) Schematic of electron beam irradiation on 2H (semiconducting) phase MoS_2_ to trigger the transition of 1T (metallic) phase triangular MoS_2_ quantum dots, where $L$ is the lattice spacing (or pitch) and $a$ is the side length of the 1T phase triangle. (**b**) Raman spectra before and after electron beam irradiation. The inset shows the partially enlarged view of the E^1^_2g_ peaks and A_1g_ peaks. As the electron beam irradiation dose increases, the intensities of E^1^_2g_ peak at 381.56 cm^−1^ and A_1g_ peak at 404.82 cm^−1^ decrease, and E^1^_2g_ peak moves to lower wavenumbers and A_1g_ peak moves to higher wavenumbers, which is different from the Raman peaks of MoS_2_′s defects^24, 25^. The intensities of the three peaks at 151.58 cm^−1^, 227.99 cm^−1^, and 305.02 cm^−1^ increase, which is the signature of the 1T phase MoS_2_^26–28^. (**c**) Cross-sectional view of the periodic quantum well, where *E*_*C*_ is the conduction band minima, *E*_*V*_ is the valance band maxima, *E*_*g*_ is the bandgap. (**d**) Two-dimensional periodic finite potential well model for calculating the emerging bandgap from quantum dot superlattice. The bottom of the triangular well is 1T phase MoS_2_. The top of the triangular potential barrier is the conduction band of 2H phase MoS_2_. ${U}_{e}$ is the height of the potential well. (**e**) Calculated band diagram of the first electron band for $a\,=\,2\,$nm and $L\,=\,4\,$nm.

Figure 1a shows the schematic of the triangular quantum dot superlattice fabricated by electron beam irradiation, where *a* is the side length of the 1T phase triangle and *L* is the lattice spacing (or pitch). The 1T/2H interface preferably forms along zigzag direction according to the observations in ref. 21, Hence, the 1T phase quantum dots are triangular. Figure 1b shows the Raman spectra of the MoS_2_ sample with different irradiation dose. As the irradiation dose increases, samples have lower E^1^_2g_ and A_1g_ peaks, and the A_1g_ peak moves to higher wavenumbers. Comparing with the Raman peaks of defects in MoS_2_, where the A_1g_ peak moves to lower wavenumbers^24, 25^, it’s clear that the change of Raman spectrum does not arise from defects, which is further discussed in Supplementary Information S2. Three new peaks at 151.58 cm^−1^, 227.99 cm^−1^, and 305.02 cm^−1^ emerge as the irradiation dose increases, which is the signature of the 1T phase MoS_2_^22, 26–28^. To further characterize the atomic structure of electron beam irradiated MoS_2_, Selected Area Electron Diffraction (SAED) measurement is conducted as shown in Supplementary Information S3. Two-dimensional Schrödinger equation, combined with Bloch theory, is employed to calculate the band structure of the 1T/2H MoS_2_ superlattice. The 1T phase MoS_2_ is metallic with zero bandgap, so it forms a quantum well for both electrons and holes with finite potential barrier as shown in Fig. 1c. The band alignment is calculated employing density functional theory (DFT), as discussed in the Supplementary Information S4, with which we find the work function of 1T phase MoS_2_ to be 5.14 eV, the work function of 2H phase MoS_2_ to be 5.09 eV, and the bandgap of 2H phase MoS_2_ to be 1.82 eV. Hence, the 1T-MoS_2_ forms electron and hole quantum wells with 0.915 eV and −0.905 eV barrier heights, respectively. The electron a﻿﻿n﻿d hole effective masses of 2H-MoS_2_ are 0.54 and 0.44 times the free electron mass, respectively^29^. The electron and hole effective masses of 1T-MoS_2_ are 0.29 and 0.23 times the free electron mass, respectively, which is discused in Supplementary Information S5. Figure 1d shows the schematic of the potential energy model for electron with barrier height ${U}_{e}\,=\,0.915\,\mathrm{eV}$, and $a$, $L$ are the same as in Fig. 1a. Fig. 1d is the calculated electron band structure for $a\,=\,2\,$nm and $L\,=\,4\,$nm. The details of the calculation method are described in the Supplementary Information S5.

## Discussion

**Figure 2:**
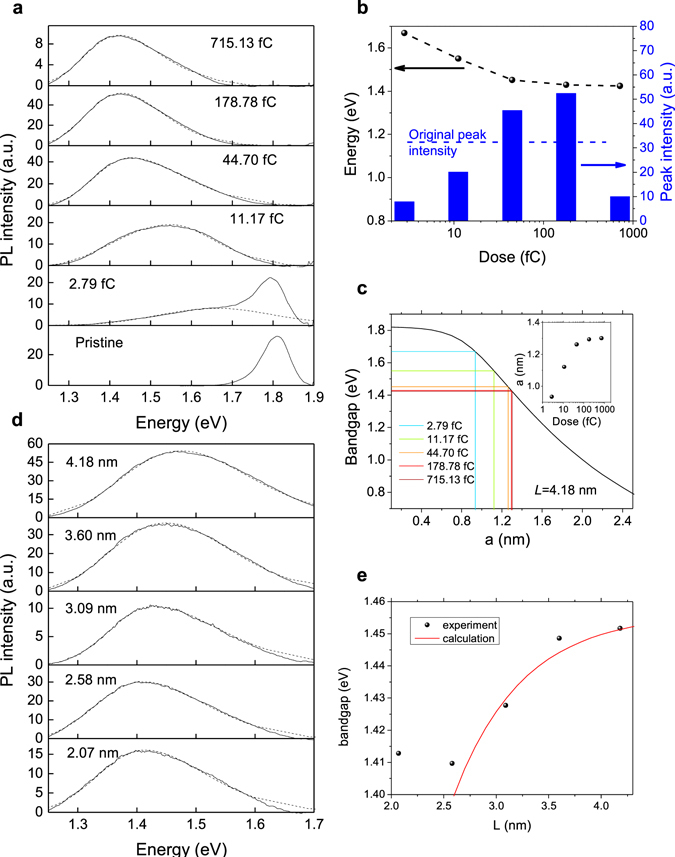
PL measurement of electron beam irradiated MoS_2_ with varying irradiation dose and lattice spacing. (**a**) PL spectra of electron beam irradiated MoS_2_ measured with 632.81 nm (1.96 eV) laser with 150.14 kW/cm^2^ power density for the samples with different irradiation doses, where the dashed lines are the fitted peaks. As the electron irradiation dose increases, the quantum dot size increases^21^. Referring to (**c**), the bandgap reduces. (**b**) The extracted peak energy and peak intensity as a function of irradiation dose from the dashed lines in (**a**), where the black dots connected with the dashed line correspond to the left axis, and the blue bars correspond to the right axis. As the irradiation dose increases, the PL peak moves towards smaller energies. The horizontal dashed line shows the peak intensity of the original peak from pristine MoS_2_. The samples with 44.70 fC and 178.78 fC irradiation dose have higher peak intensity than that of the pristine sample. (**c**) Calculated bandgap as function of quantum dot size $a$ with $L\,=\,$4.18 nm. The colored vertical and horizontal lines mark measured peak energies and the corresponding quantum dot sizes $a$. The inset is the extracted quantum dot size $a$ as function of irradiation dose. By controlling the irradiation dose, sub-nanometer precision can be achieved. As the irradiation dose increases, the estimated size $a$ progresses towards 1.3 nm (1.5 nm in diameter), which is limited by the size of the focused electron beam (2 nm). (**d**) PL spectra of electron beam irradiated MoS_2_ measured with 632.81 nm (1.96 eV) laser with 150.14 kW/cm^2^ power density for the samples with different lattice spacing $L$ but identical point irradiation dose of 44.70 fC, where the dashed lines are the fitted peaks. (**e**) The extracted peak energy (bandgap) as a function of spatial distance $L$ from the dashed lines in (**d**). The red curve corresponds to calculated results using $a\,=\,$1.26 nm from data in (**c**). Note that the precision of the spatial distance, $L$, is limited by the resolution of the irradiation system.

In order to characterize the new bandgap, the irradiated MoS_2_ samples are examined by PL spectrometer with 632.81 nm (1.96 eV) wavelength laser. Note that, before every measurement, 698.88 kW/cm^2^ laser pulse is applied to the sample, in order to anneal the sample to reduce the surface moisture, which may reduce the PL signal. Figure 2a shows the PL spectra on different MoS_2_ samples having quantum dot arrays with different irradiation dose for $L\,=\,4.18\,$nm, where the dashed lines are fitted peaks with the bi-Gaussian method^30^. The pristine MoS_2_ exhibits a PL peak at 1.81 eV in agreement with its bandgap. After electron beam irradiation, the original PL peak reduces and the new PL peak emerges. As the irradiation dose increases, the original PL peak vanishes and the new PL peak progresses towards 1.4 eV. Such a large red-shift is not observed in any defect induced PL peaks^24, 25^. The fitted peak energy and peak intensity as function of irradiation dose are shown in Fig. 2b. Surprisingly, the new PL peak intensity increases and becomes higher than the original PL peak when the irradiation doses are 44.70 fC and 178.87 fC. This phenomenon could be due to the fact that lower bandgap of quantum dots attracts non-radiative charges from defects, which increases the radiative recombination. However, with even higher dose of 715.13 fC, the PL peak intensity reduces, which may result from electron irradiation induced damage. Moreover, according to Lin *et al*.^21^, higher irradiation dose can increase the quantum well size $a$, and as shown in Fig. 2c, the quantum mechanical calculation shows lower bandgap with larger $a$. (The calculated band edges for electrons and holes are specifically shown in the Supplementary Information S5, Fig. S8). Projecting the PL peak energy to the calculated bandgap as shown in Fig. 2c, the corresponding quantum dot triangular side length $a$ can be estimated, as shown in the inset. For irradiation dose ranging from 2.79 fC to 715.13 fC, the peak energies change from 1.67 eV to 1.42 eV and $a$ changes from 0.93 nm to 1.30 nm (1.0 nm to 1.5 nm in diameter) with sub-nanometer precision. The minimum area of the quantum dot observed in ref. 21 is 1.08 nm^2^ (or *a* $\,=\,$0.94 nm), which agrees well with our estimation. Note that, the focused electron beam has a Gaussian-like intensity distribution, and only the region around the center of the electron beam has enough intensity to trigger 1T phase transition. So the size of the quantum dot is always smaller than the focused electron beam size (2 nm in our case), which is also discussed in Supplementary Information S1. As shown in the inset of Fig. 2c, as the irradiation dose increases, the estimated size *a* progresses towards 1.3 nm (1.5 nm in diameter), which is limited by the size of the focused electron beam. Another parameter that can be precisely controlled is the lattice spatial distance $L$. Figure 2d shows the PL spectra on different regions with different lattice spatial distances *L* for the same point radiation dose of 44.70 fC. The result is summarized in Fig. 2e. As the $L$ increases, the PL peak energy increases. The red curve in Fig. 2e is the calculated bandgap as function of $L$, where $a$ is assumed to be 1.26 nm, which is extracted from Fig. 2c. (The calculated band edges for electrons and holes are specifically shown in the Supplementary Information S5, Fig. S9). For *L* larger than 2.07 nm, the curve fits well with the measured data. For *L* $\,=\,$2.07 nm, since the focused electron beam has resolution of about 2 nm as shown in the Supplementary Information S1, the fluctuation of electron beam may produce unpredictable results, which may explain why the data is off the calculated red line.

**Figure 3:**
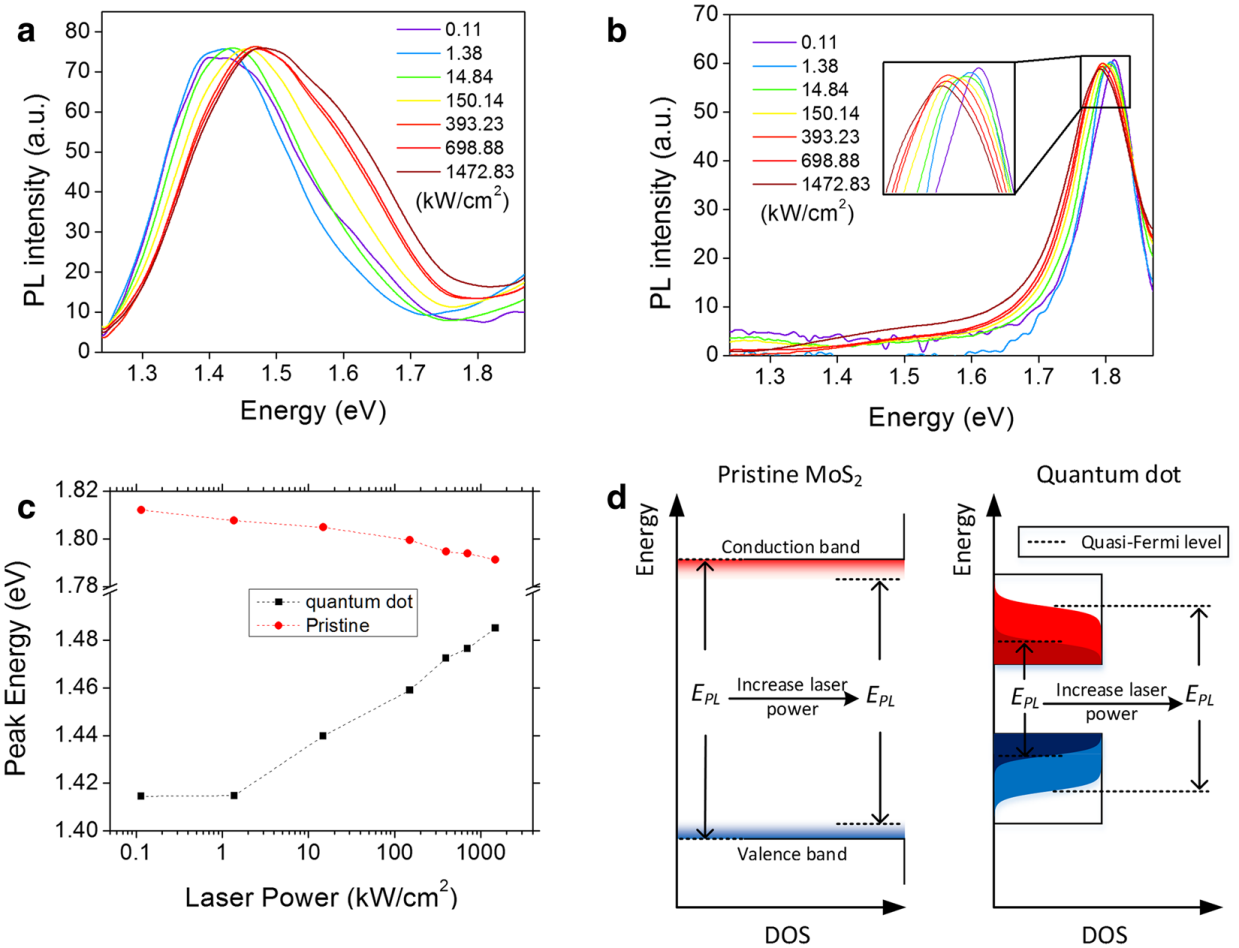
PL measurement of electron beam irradiated MoS_2_ with varying laser power. (**a**) The PL spectra for the same MoS_2_ sample with 4.18 nm lattice spacing and 44.70 fC electron irradiation dose under different laser power. (**b**) The PL spectra for pristine MoS_2_ sample under different laser power. The data in (**a**) and (**b**) are normalized by bringing the peak intensity to the same value for ease of comparison. All the original data are available in the Supplementary Information S6 (Fig. S10). (**c**) Peak energy for the data in (**a**) and (**b**) as a function of pumping laser power. (**d**) Schematic to explain the effect of increasing laser power. For pristine MoS_2_, higher laser power can induce higher temperature, which can decrease the bandgap according to Varshni theory^31, 32^. However, the quantum dot samples show larger bandgap at larger laser power. This is because the quantum dots have smaller density of states (DOS) near the band edges than that of pristine MoS_2_, and larger laser power can generate larger number of electrons and holes, which increases the separation between the quasi-Fermi levels of electrons and holes. (Black rectangular regions indicate DOS; red/blue shades show electron/hole distributions, respectively).

Another interesting finding is the blue-shift (peak energy shifts towards higher energies) of the PL peak of the electron beam irradiated sample under high pumping laser power. As shown in Fig. 3a and b, as the laser power increases, the PL peaks of the pristine MoS_2_ move towards smaller energy values, however, the PL peaks for the quantum dot array on the electron irradiated MoS_2_ move towards larger energy values. The peak energy as a function of laser power is plotted in Fig. 3c. As illustrated in Fig. 3d, for the pristine sample, higher pumping laser power induces higher temperature, which broadens the band edges and reduces the bandgap as described by Varshni theory^31, 32^. On the other hand, the quantum dots form confined modes on MoS_2_, which have a lower density of states (DOS) than pristine MoS_2_. Higher pumping laser power generates more electrons and holes, so the electrons’ and holes’ quasi-Fermi levels will have large separation. This effect overpowers the temperature induced bandgap reduction as evidenced by a significant blue-shift toward higher energies. For laser power lower than 1.38 kW/cm^2^, the small amount of electrons and holes can only fill the states at the bottom of the conduction band and the top of the valence band of the quantum well superlattice, respectively. Hence, the blue-shift is not significant.

**Figure 4:**
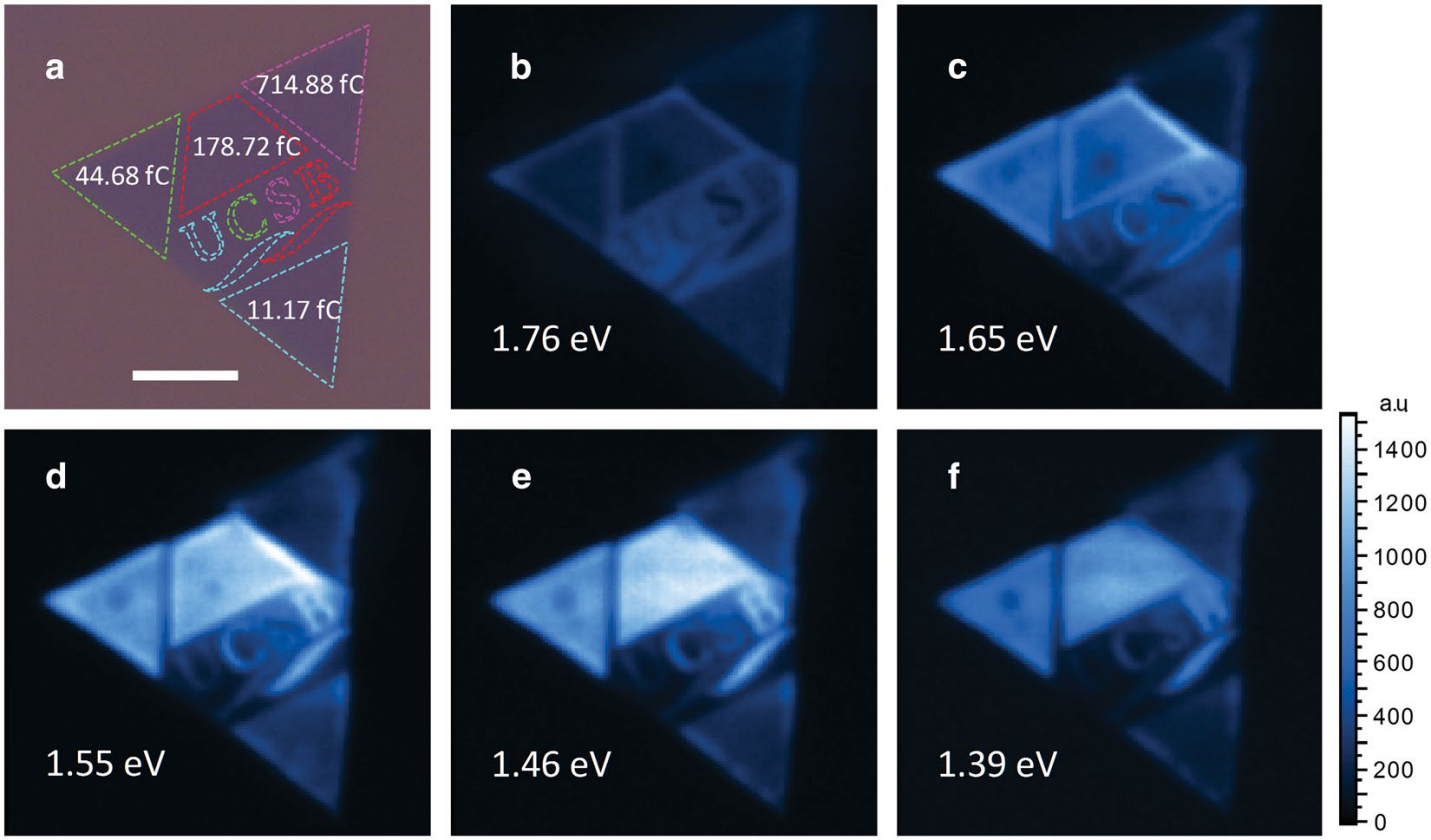
PL mapping on electron beam irradiated sample with a designed pattern under 632.81 nm laser with 1472.83 kW/cm^2^ laser power. (**a**) The optical microscope image of the sample overlapped with the designed pattern with $L\,=\,$4.18 nm lattice spacing. The colors of the dashed lines encompassing the four regions (and in “UCSB”), blue, green, red and pink correspond to 11.17 fC, 44.68 fC, 178.72 fC, and 714.88 fC, respectively. (**b**–**f**) are the PL mapping for 1.76 eV, 1.65 eV, 1.55 eV, 1.46 eV, and 1.39 eV, respectively. The white scale bar is 10 $\mu$m.

In order to provide direct evidence of large-scale quantum dot array and to verify the uniformity and controllability of the electron beam irradiation induced new PL peaks, PL mapping is conducted on a monolayer MoS_2_ sample having quantum dot arrays (~30 μm × 30 μm) made with different irradiation dose under 632.81 nm laser with 1472.83 kW/cm^2^ laser power, as shown in Fig. 4. In Fig. 4a, the different colors of the dashed lines correspond to different irradiation doses from 11.17 fC to 714.88 fC. Figure 4b to 4f show the slices of PL mapping at different energies with the energy spectrum ranging from 1.39 eV to 1.76 eV. For example, at 1.76 eV, the pristine region (region around “UCSB”) lights up and the electron beam irradiated regions remain dark. At 1.65 eV, the letter “U” and the bottom triangle, whose irradiation doses are 11.17 fC, reach their brightest in comparison to the same regions in other figures. At 1.55 eV, the letter “C” and the left triangle, whose irradiation doses are 44.68 fC, reach their brightest. At 1.46 eV, the letter “B” and the top trapezoid, whose irradiation doses are 178.72 fC, reach their brightest. The letter “S” and right triangle, whose irradiation doses are 714.88 fC, reach the brightest at 1.39 eV. It’s interesting to note that, in Fig. 4c, the edges of the trapezoid are brighter than the center, which is contributed by the charges from the surrounding pristine region.

In summary, we have demonstrated the fabrication of 1T phase MoS_2_ quantum dot superlattice on monolayer single crystal 2H phase MoS_2_ by focused electron beam irradiation. The size of the quantum dots can be tuned by the irradiation dose with sub-nanometer precision, and the location of the quantum dots can be designed with nanometer precision, which depends on the resolution of the electron beam system (2 nm in this work). The scale of the quantum dot arrays is only limited by the size of the electron beam lithography machine. By designing the size of the quantum dots and the lattice spacing of the quantum dot superlattice, the bandgap of the monolayer MoS_2_ can be tuned over a range from 1.81 eV (for pristine monolayer MoS_2_) to 1.42 eV. Moreover, the quantum dot superlattice on MoS_2_ exhibits brighter PL than that of pristine MoS_2_ with 44.68 fC and 178.72 fC irradiation dose and shows blue-shift while increasing the pumping laser power. The ability to control the quantum dots with such precision opens up new pathways for scalable quantum computers and quantum photon sources. Moreover, our idea of creating quantum dot superlattice on 2D materials can inspire the design of a completely new suite of *artificial 2D-crystals*. While the tunable direct band gap could usher in a new generation of light-emitting devices for photonics applications.

## Electronic supplementary material

Supplementary Info
