# Supplementary material for: Designing artificial 2D crystals with site and size controlled quantum dots
Source: Sci Rep. 2017 Aug 30;7:9965. doi: 10.1038/s41598-017-08776-3 (PMC5577271; doi:10.1038/s41598-017-08776-3)
Supplement: Supplementary file 1 — Supplementary Info [file 41598_2017_8776_MOESM1_ESM.pdf]

Supplementary Information

Designing artificial 2D crystals with site and size controlled quantum dots

Xuejun Xie[1], Jiahao Kang[1], Wei Cao[1], Jae Hwan Chu[1], Yongji Gong[2], Pulickel M. Ajayan[2] and Kaustav Banerjee[1]*

[1]Department of Electrical and Computer Engineering, University of California, Santa Barbara, California 93106, United States

[2]Department of Materials Science and Nanoengineering, Rice University, Houston, Texas 77005, United States

*Correspondence to: kaustav@ece.ucsb.edu

S1. Electron beam and quantum dot size estimation

The resolution of FEI XL-30 SIRION Scanning Electron Microscope (SEM) is 2 nm at 30 kV on spot number 4 provided by FEI company. Since we are using SEM for electron beam irradiation, the focus quality can affect the electron beam size, and the resolution of the SEM image can be used to estimate the real size of the electron beam. As shown in Fig. S1, on spot number 4 at 30 kV, the resolution of the SEM is about 2 nm.

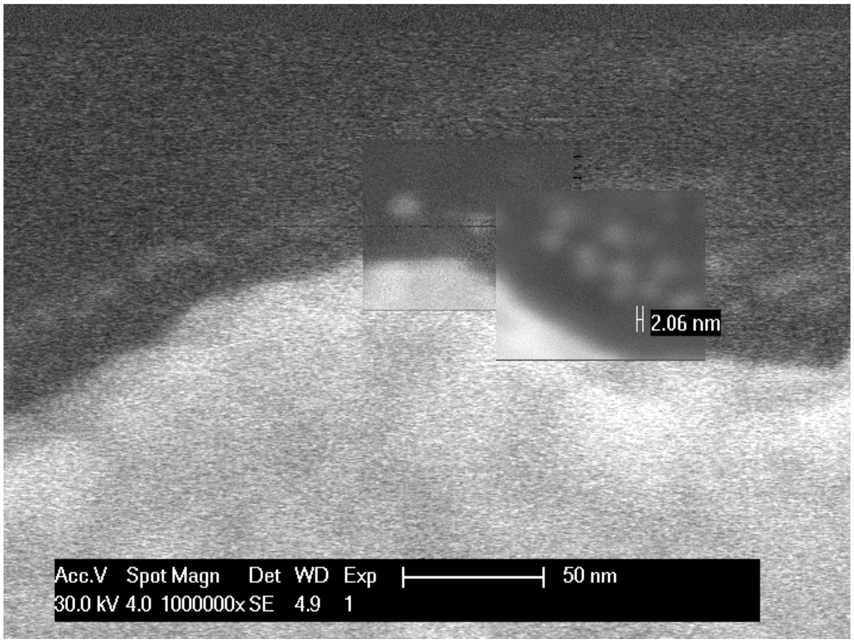

Figure S1. SEM image was taken under the same condition as that used for electron beam radiation except for the magnification. The minimum distinguishable feature size is about 2 nm.

Because the focused electron beam has a Gaussian-like intensity distribution, only the region around the center of the electron beam has enough intensity to trigger 1T-phase transition. Hence, as illustrated in Fig. S2, the size of the quantum dot (QD) should always be smaller than the focused electron beam size. In our case, the size of our focused electron beam is around 2 nm. As a result, the estimated size of our quantum dots ranges from 0.93 nm to 1.3 nm (in terms of the length of the edge (a) of the triangle for 1T-phase QD), or 1.0 nm to 1.5 nm (in terms of the diameter of the circle that encompasses the triangle). The minimum area of the quantum dot observed in Ref. [21] (in the main article) is 1.08 nm^2 (or a = 0.94 nm), which agrees well with our estimation.

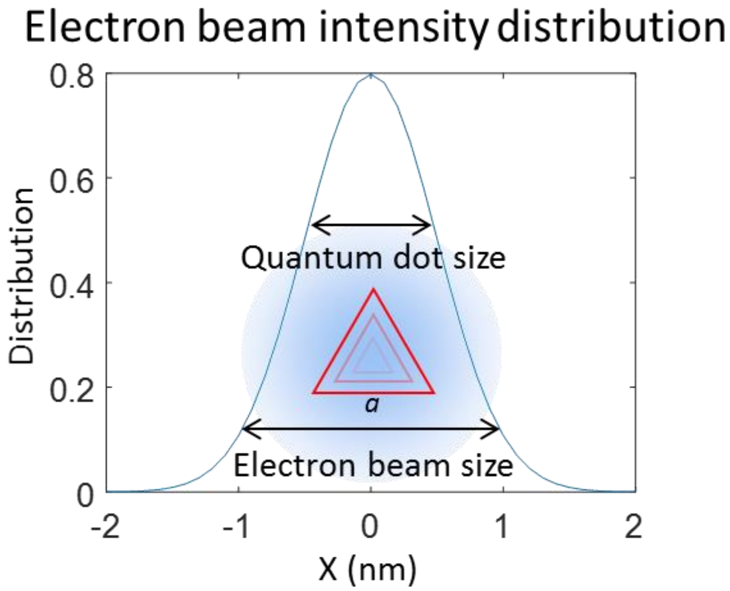

Figure S2: This figure explains the reason why the size of the quantum dot is smaller than the size of the focused electron beam. The blue circle represents the electron beam; the red triangle represents the quantum dot. The plot is the Gaussian distribution of the electron beam intensity for a 2 nm electron beam. Since the center of the electron beam has maximum intensity, the quantum dot grows from the center of the electron beam. Hence, the quantum dot is always smaller than focused electron beam.

S2. Raman and PL spectrum of defects in MoS_2

The Raman beam spot, which covers many e-beam irradiated spots, is used to monitor the material properties within a relatively large region, instead of a single e-beam irradiated spot. Any change to this 2H- phase region, induced by e-beam, will be reflected as new peaks in Raman spectrum. We note that defects in MoS_2 give rise to completely different Raman spectrum and PL spectrum as demonstrated by Tongay *et al*.[1] and Philippe K. Chow *et al.*[2] We provide below the Raman spectrum and PL spectrum data reported in those papers (Fig. S3 (a) & (b)) in comparison with our data (Fig. S3 (c)).

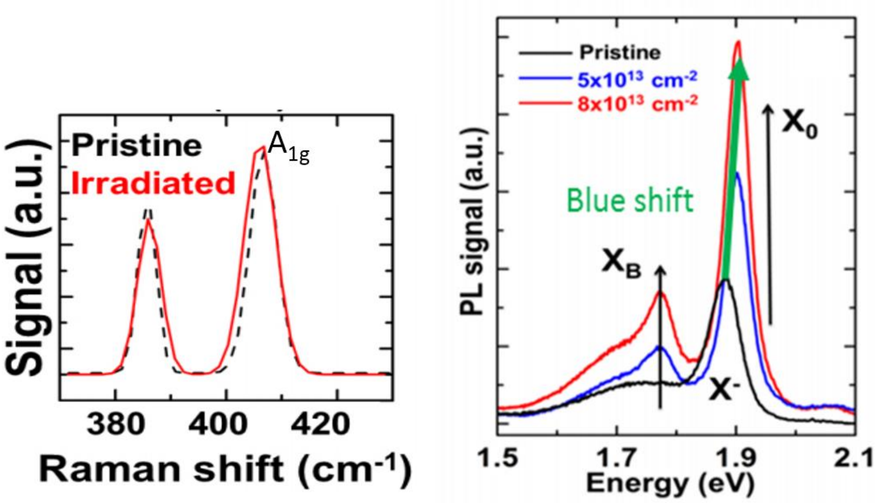

Figure S3: (a) Raman spectrum (left) and PL spectrum (right) of defects in MoS_2 by alpha particle irradiation in Tongay *et al*.[1] The A_{1g} peak moves to lower wavenumbers after irradiation. As the irradiation dose (energy) of alpha particle increases, the X_0 peak shows blue shift and the X_B peak shows no shift.

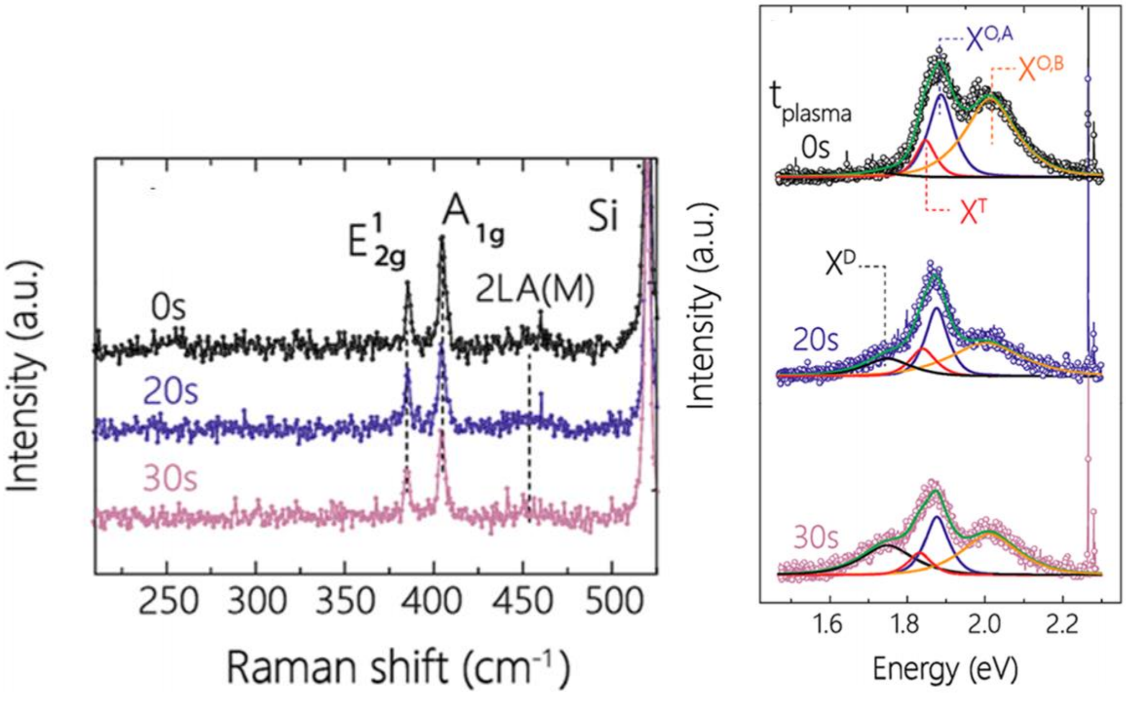

Figure S3: (b) Raman spectrum (left) and PL spectrum (right) of defects in MoS_2 by Argon plasma treatment in Philippe K. Chow *et al*.[2] The A_{1g} peak moves to lower wavenumbers after treatment. No new peaks are observed. As the plasma treatment time increases, the PL peak shows no shift.

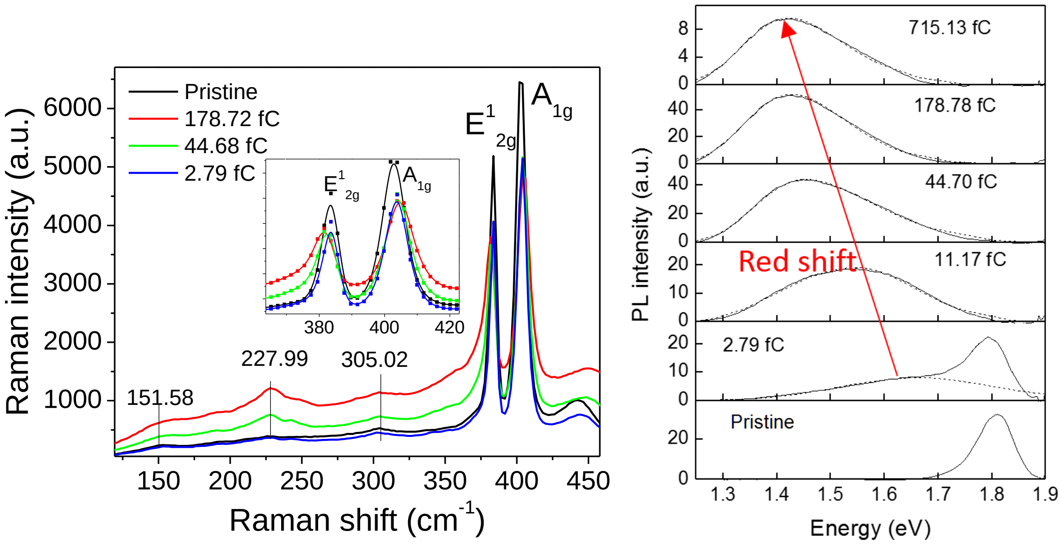

Figure S3: (c) Raman spectrum (left) and PL spectrum (right) of quantum dot superlattice on MoS_2 in our work. The A_{1g} peak moves to higher wavenumbers after irradiation and three new peaks at 151.58 cm^{-1}, 227.99 cm^{-1}, and 305.02 cm^{-1} emerge. As the irradiation dose (energy) of focused electron beam increases, the PL peak shows a large shift towards 1.4 eV (Red shift). Such a big shift towards lower peak energy is not exhibited by any MoS_2 defects.

S3. TEM SAED characterization of focused electron beam irradiated MoS_2

At first, we performed STEM measurements. However, given the unstable nature of the quantum dots, high energy focused electron in STEM can agitate the quantum dots and anneal the sample. As a result, it's very hard to observe the 1T phase MoS_2 without annealing them back into 2H phase MoS_2. Therefore, we can not observe any 1T phase MoS_2 under high resolution STEM. Subsequently, we performed Selected Area Electron Diffraction (SAED) measurement as shown in Fig. S4. The sample is first irradiated with focused electron beam on SiO_2 substrate, then transferred to TEM grid as shown in Fig. S4d. The PL measurement on the MoS_2 on TEM grid as shown in Fig. S4e confirms the success of sample preparation. Fig. S4c shows the TEM imaging. The irradiated region is brighter than the pristine region. This could be due to the electron beam induced cleaning of surface contamination. The intensity of the first order peaks of SAED data shown in Fig. S4a,b is summarized in Fig. S4g. According to this paper,[3] 1T phase MoS_2 shows larger difference between strong peaks and week peaks. As illustrated in Fig. S4f, for the sample with 1T phase quantum dot, the 1T phase and 2H phase MoS_2 have aligned strong and weak diffraction direction as defined in this paper.[3] So the 1T phase quantum dots can increase the difference between strong and weak peaks. The larger % difference in the irradiated sample as shown in Fig. S4g is the direct structural evidence of the existence of 1T phase. On the other hand, 1T phase MoS_2 has larger SAED peak wavenumber comparing to 2H phase MoS_2.[4] As shown in Fig. S4h, the SAED peaks of Focused electron irradiated MoS_2 shows larger wavenumber comparing to pristine 2H phase MoS_2. This is another structural evidence of 1T phase MoS_2.

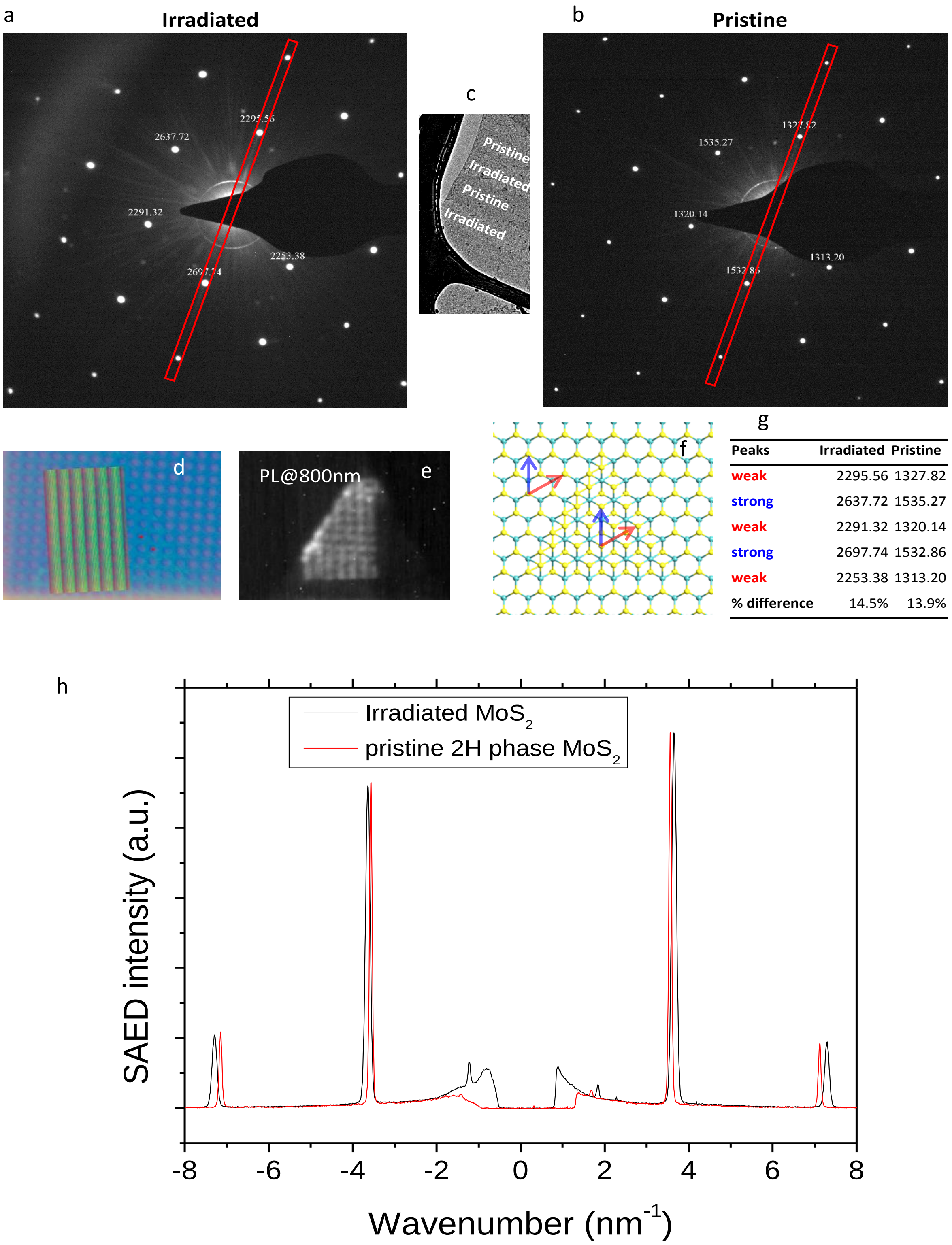

Peaks	Irradiated	Pristine
weak	2295.56	1327.82
strong	2637.72	1535.27
weak	2291.32	1320.14
strong	2697.74	1532.86
weak	2253.38	1313.20
% difference	14.5%	13.9%

Figure S4: **(a)** SAED for focused electron beam irradiated MoS_2. (b) SAED for pristine MoS_2. **(c)** TEM image of the sample. **(d)** Optical image of the MoS_2 sample on TEM sample grid with overlapping of the designed irradiation regions. **(e)** PL mapping of the MoS_2 on TEM grid at 800 nm with 633 nm laser. **(f)** Schematic illustration of the electron scattering condition. One arrow shows one of the directions of the scattering, which corresponds to one of

the bright spots in SAED. Blue arrow shows the direction with stronger scattering and red arrow shows direction with weaker scattering as discussed in this paper.[3] **(g)** Summary of the first order peak intensities. The "% difference" is calculated using [average(strong) – average(weak)]/average(strong). 1T phase MoS_2 exhibits larger % difference.[3] **(h)** The SAED plots are extracted from the data in (a) that are marked with the red rectangle. The SAED peaks of focused electron irradiated MoS_2 show larger wavenumbers compared to those of pristine 2H phase MoS_2. This is another structural evidence of 1T phase MoS_2.[4]

S4. Band structures and work functions of 1T and 2H phase MoS_2

The band structures and the work functions of 1T and 2H phase MoS_2 were calculated via ab-initio density functional theory (DFT). Local density approximation (LDA) was adopted for the exchange correlations[5], which provides consistent accuracy for band structure calculations of TMDs.[6] A double-ζ polarized basis set was used for expanding electronic density. The calculations were performed using Atomistix ToolKit (ATK).[7] 9×9×1 k-points were sampled in the Brillouin zone. The temperature was set to be 300 K. The density mesh cut-off was 75 Rydberg and the maximum force was 0.05 eV/Å for geometry optimization (relaxation). Vacuum was added both on the top and on the bottom of the MoS_2 monolayer, to ensure that the effective potential has enough distance to decay to the vacuum level and no basis functions extend to the edge of the cell, respectively.

The band structures of 1T and 2H MoS_2 are shown in Fig. S5. The effective masses were thus calculated based on the curvature of the dispersion curve at the extrema near the Fermi level using the classical equation, $m^* = \hbar^2/k \times 1/(\partial^2 E/\partial k^2)$. For each phase, the lowest effective mass was taken for each type of carriers (electrons and holes) for simplicity although there can be more than one extrema near the Fermi level.

For work function calculation, an additional layer of MoS_2 atoms with basis sets but without any pseudopotential core or charge were put on top of the MoS_2 surface. The basis set orbitals can be populated in order to host a finite electron density in a region where there are no real atoms, which extends the range of the electron density into the vacuum so that it has time to decay. The effective potential is normalized to zero on the top boundary (far away from the surface) of the unit cell by using the Dirichlet boundary condition. The work function then becomes equal to the Fermi level or chemical potential. The gradient of the potential is made to vanish on the bottom of the system by Neumann condition. The above setup provides accurate results for many crystals compared with experiments.[8]

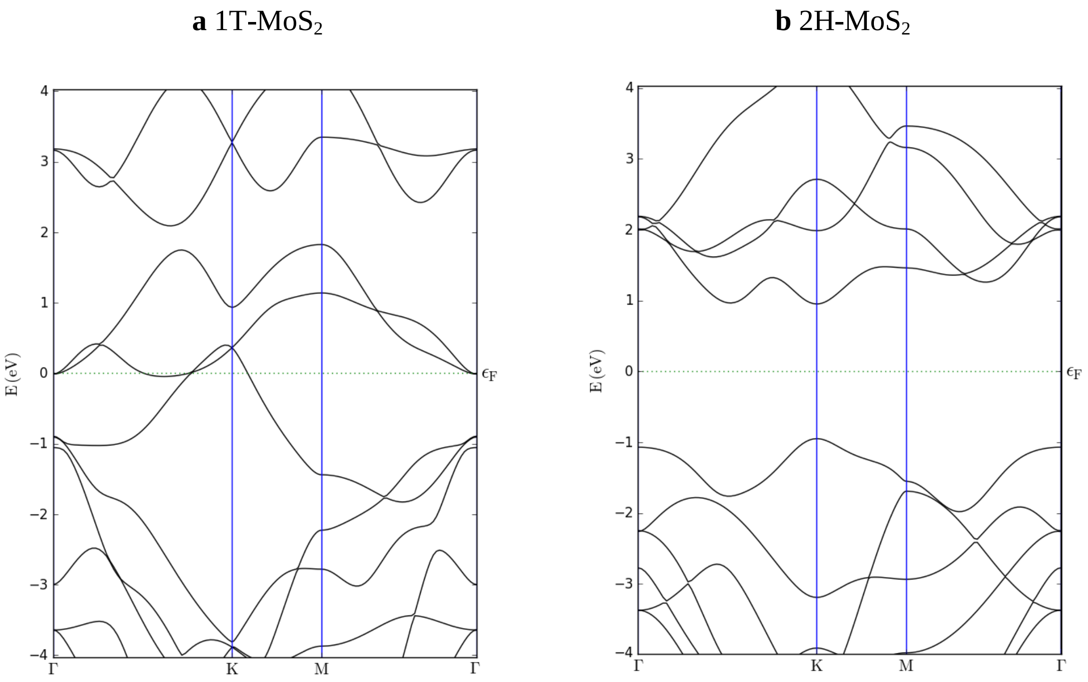

Figure S5: Calculated band structures of **(a)** 1T and (**b)** 2H phase MoS_2 by DFT.

S5. Band structure of 1T-2H superlattice

In this section, we first discuss the band structure of 1T-2H phase superlattice qualitatively, then calculate it quantitatively. Please note that we do not need to assume any specific 1T-MoS_2 quantum dot size or electron/hole mass to explain the red-shifted PL (Fig. 2a in main article). This is due to the following reasons. First off, any quantum well will always have lower ground state energy w.r.t the height of the potential well. As a result, the effective bandgap of the metallic quantum dot will be smaller w.r.t that of the surrounding semiconductor (as illustrated in Fig. S6).

Moreover, because larger quantum dot has smaller ground state energy, the metallic quantum dot's bandgap always decreases with increasing size of the quantum dot (see Fig. S6). Hence, "red shift" does not depend on the specific size of the quantum dot.

Additionally, the above conclusion is independent of the specific values of the electron/hole mass. Therefore, the explanation for the red-shift does not depend on the effective mass used in the following calculations.

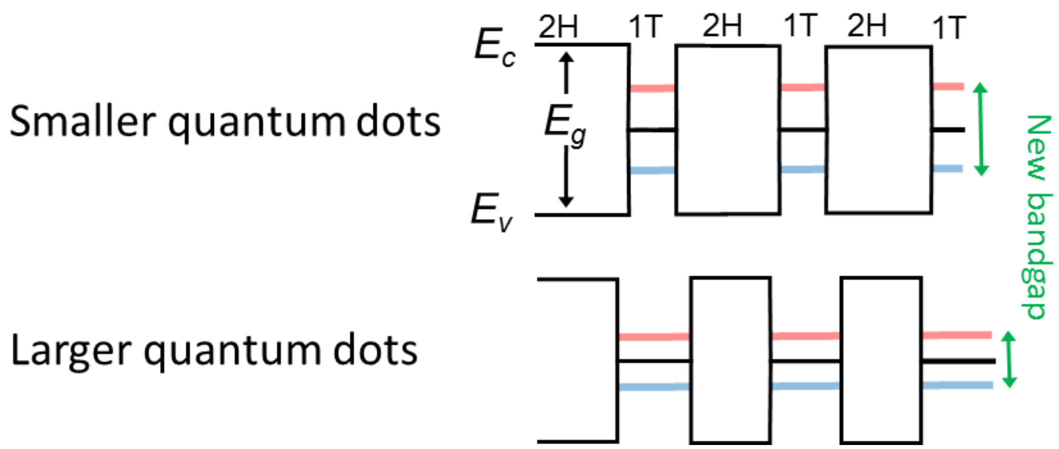

Figure S6: Band diagrams for metallic 1T-phase quantum dot superlattice in semiconducting 2H-phase MoS_2. The E_g, E_c, E_v are the bandgap, conduction band, valence band of 2H-phase MoS_2, respectively. The red line and blue line are the conduction band and valence band of the quantum dot superlattice, respectively. The energy difference between blue and red line is the new bandgap as indicated by green arrows.

The derivation starts from the two-dimensional Schrödinger Equation,

$$-\frac{\hbar^2}{2m^*}\Delta_{\boldsymbol{r}}\psi(\boldsymbol{r}) + U(\boldsymbol{r})\psi(\boldsymbol{r}) = \varepsilon\psi(\boldsymbol{r}) \tag{1}$$

Where m^* is the effective mass (for 2H phase MoS_2 electron $m^*_{e\_2H} = 0.54m_e$ or hole $m^*_{h\_2H} = 0.44m_e$, for 1T phase MoS_2 electron $m^*_{e\_1T} = 0.29m_e$ or hole $m^*_{h\_1T} = 0.23m_e$), $\Delta_{\boldsymbol{r}} = \nabla^2_{\boldsymbol{r}} = \left(\frac{\partial}{\partial x} + \frac{\partial}{\partial y}\right)^2$ is the Laplacian operator, $\psi(\boldsymbol{r})$ is the wave function at vector $\boldsymbol{r} = x\hat{\boldsymbol{x}} + y\hat{\boldsymbol{y}}$, ε is the eigenvalue, and $U(\boldsymbol{r})$ is the potential energy at $\boldsymbol{r}$,

$$U(\boldsymbol{r}) = \begin{cases} 0 & (\boldsymbol{r}\ in\ QW) \\ U_e\ or\ U_h & (\boldsymbol{r}\ out\ of\ QW) \end{cases} \tag{2}$$

Where U_e and U_h are the potential barrier heights of electrons and holes, respectively. According to the DFT simulations, $U_e = 0.915$ eV and $U_h = -0.905$ eV. The $U(\boldsymbol{r})$ is defined by the triangle quantum dot structure as shown in Fig. S7a and illustrated in Fig. S7b.

$\varphi(\boldsymbol{r})$ is a periodic function, and the boundary conditions can be expressed as:

$$\psi(\boldsymbol{r}) = \psi(\boldsymbol{r})\exp\left(i\boldsymbol{k}\cdot(n_x L\hat{\boldsymbol{x}} + n_y L\hat{\boldsymbol{y}})\right), n_x = 0, 1, 2, \dots, n_y = 0, 1, 2, \dots \tag{3}$$

where L is the period of the super lattice, $\hat{\boldsymbol{x}}$ and $\hat{\boldsymbol{y}}$ are the unit basis, and $\boldsymbol{k} = k_x\hat{\boldsymbol{x}} + k_y\hat{\boldsymbol{y}}$ is the wave vector.

The problem can be discretized. First let the real space (*x*-*y* plane) be sampled by $N \times N$ points, by assuming:

$$\boldsymbol{r} = x\hat{\boldsymbol{x}} + y\hat{\boldsymbol{y}} = \frac{n_x}{N} L\hat{\boldsymbol{x}} + \frac{n_y}{N} L\hat{\boldsymbol{y}};\ n_x, n_y = 1, 2, \dots, N \tag{4}$$

For convenience, we denote $U(\boldsymbol{r})$ and $\psi(\boldsymbol{r})$ as $U(n_x, n_y)$ and $\psi(n_x, n_y)$.

Assume an $N^2 \times 1$ matrix Φ is in the form of:

$$\Phi = \begin{bmatrix} \Phi_1 \\ \Phi_2 \\ \vdots \\ \Phi_{N^2-1} \\ \Phi_{N^2} \end{bmatrix} = \begin{bmatrix} [\psi(1,1)\ \ \psi(2,1)\ \dots\ \psi(N-1,1)\ \ \psi(N,1)]^T \\ [\psi(1,2)\ \ \psi(2,2)\ \dots\ \psi(N-1,2)\ \ \psi(N,2)]^T \\ \vdots \\ [\psi(1,N)\ \ \psi(2,N)\ \dots\ \psi(N-1,N)\ \ \psi(N,N)]^T \end{bmatrix} \tag{5}$$

The Schrödinger Equation can then be discretized as $\mathrm{H}\Phi = \mathrm{E}\Phi$. Here $\mathrm{H} = \mathrm{H}_0 + \mathrm{U}$ is the Hamiltonian matrix. Then,

$$\mathrm{H}_0\Phi + \mathrm{U}\Phi = -\frac{\hbar^2}{2m^*}[\Delta_{\boldsymbol{r}}]\Phi + \mathrm{U}\Phi\ = \mathrm{E}\Phi \tag{6}$$

where $\mathrm{H}_0 = -\frac{\hbar^2}{2m^*}[\Delta_{\boldsymbol{r}}]$, $[\Delta_{\boldsymbol{r}}]$ is the Laplacian matrix, U is an $N^2 \times N^2$ matrix:

$$\begin{aligned} \mathrm{U} &= diag\left(\begin{bmatrix} \mathrm{U}_1 \\ \mathrm{U}_2 \\ \vdots \\ \mathrm{U}_{N^2-1} \\ \mathrm{U}_{N^2} \end{bmatrix}\right) \\ &= diag\left(\begin{bmatrix} [U(1,1)\ \ U(2,1)\ \dots\ U(N-1,1)\ \ U(N,1)]^T \\ [U(1,2)\ \ U(2,2)\ \dots\ U(N-1,2)\ \ U(N,2)]^T \\ \vdots \\ [U(1,N)\ \ U(2,N)\ \dots\ U(N-1,N)\ \ U(N,N)]^T \end{bmatrix}\right) \end{aligned} \tag{7}$$

and E is an $N^2 \times N^2$ matrix,

$$\mathrm{E} = diag\left(\begin{bmatrix} E_1 \\ E_2 \\ \vdots \\ E_{N^2-1} \\ E_{N^2} \end{bmatrix}\right) \tag{8}$$

where $E_1, E_2, \dots, E_{N^2}$ are the energy levels.

The function *diag*($V_{M\times 1}$) represents a square diagonal matrix with the elements of $M \times 1$ matrix (or vector) $V_{M\times 1}$ on the main diagonal. Now the form of vector $[\Delta_{\boldsymbol{r}}]\Phi$ should be derived. Let vector $\Theta = [\Delta_{\boldsymbol{r}}]\Phi$ and,

$$\Theta = \begin{bmatrix} \Theta_1 \\ \Theta_2 \\ \vdots \\ \Theta_{N^2-1} \\ \Theta_{N^2} \end{bmatrix} = \begin{bmatrix} [\theta(1,1)\ \ \theta(2,1)\ \dots\ \theta(N-1,1)\ \ \theta(N,1)]^T \\ [\theta(1,2)\ \ \theta(2,2)\ \dots\ \theta(N-1,2)\ \ \theta(N,2)]^T \\ \vdots \\ [\theta(1,N)\ \ \theta(2,N)\ \dots\ \theta(N-1,N)\ \ \theta(N,N)]^T \end{bmatrix} \tag{12}$$

Then,

$$\theta(n_x, n_y) = \left(\frac{1}{L/N}\right)^2 \left[\psi(n_x+1, n_y) + \psi(n_x-1, n_y) + \psi(n_x, n_y+1) + \psi(n_x, n_y-1) - 4\psi(n_x, n_x)\right] \tag{14}$$

$$\Theta = [\Delta_{\boldsymbol{r}}]\Phi = \left(\frac{1}{L/N}\right)^2 (\mathrm{A}\Phi + \mathrm{B}\Phi + \mathrm{C}\Phi + \mathrm{D}\Phi - 4\mathrm{I}\Phi) \tag{15}$$

where I is the elemental matrix. Using the boundary conditions,

$$\Phi = \Phi \exp\left(i\boldsymbol{k}\cdot(n_x L\hat{\boldsymbol{x}} + n_y L\hat{\boldsymbol{y}})\right), m_x = 0, 1, 2, \dots, m_y = 0, 1, 2, \dots \tag{16}$$

One can derive matrices A, B, C and D,

$$\mathrm{A} = \begin{bmatrix} \mathrm{A}_0 & & & \\ & \mathrm{A}_0 & & \\ & & \ddots & \\ & & & \mathrm{A}_0 \end{bmatrix}_{N^2\times N^2}, \mathrm{B} = \begin{bmatrix} \mathrm{B}_0 & & & \\ & \mathrm{B}_0 & & \\ & & \ddots & \\ & & & \mathrm{B}_0 \end{bmatrix}_{N^2\times N^2} \tag{17}$$

where,

$$\mathrm{A}_0 = \begin{bmatrix} 0 & 1 & & & \\ & \ddots & \ddots & & \\ & & \ddots & \ddots & \\ & & & \ddots & 1 \\ e^{ik_xL} & & & & 0 \end{bmatrix}_{N\times N}, \quad \mathrm{B}_0 = \begin{bmatrix} 0 & & & & e^{-ik_xL} \\ 1 & \ddots & & & \\ & \ddots & \ddots & & \\ & & \ddots & \ddots & \\ & & & 1 & 0 \end{bmatrix}_{N\times N} \tag{18}$$

and,

$$\mathrm{C} = \begin{bmatrix} \mathbf{0} & \mathbf{0} \\ \mathrm{C}_0 & \mathbf{0} \end{bmatrix}_{N^2\times N^2}, \mathrm{D} = \begin{bmatrix} \mathbf{0} & \mathrm{D}_0 \\ \mathbf{0} & \mathbf{0} \end{bmatrix}_{N^2\times N^2} \quad (19)$$

where,

$$\mathrm{C}_0 = \begin{bmatrix} e^{ik_xL} & & & \\ & \ddots & & \\ & & \ddots & \\ & & & e^{ik_xL} \end{bmatrix}_{N\times N}, \quad \mathrm{D}_0 = \begin{bmatrix} -e^{ik_xL} & & & \\ & \ddots & & \\ & & \ddots & \\ & & & -e^{ik_xL} \end{bmatrix}_{N\times N} \quad (20)$$

The eigenvalues solved from the Hamiltonian matrix H are the energy levels $E_1, E_2, \ldots, E_{N^2}$, and the eigenvectors $\Phi^{E1}, \Phi^{E2}, \Phi^{E3}, \ldots, \Phi^{EN^2}$ solved from H are the wave functions. The Φ^{E1} is plotted in Fig. S7c. The E_1 and E_2 *vs.* k, as function of a, are plotted in Fig. S8. The E_1 and E_2 *vs.* k as function of L are plotted in Fig. S9.

Results:

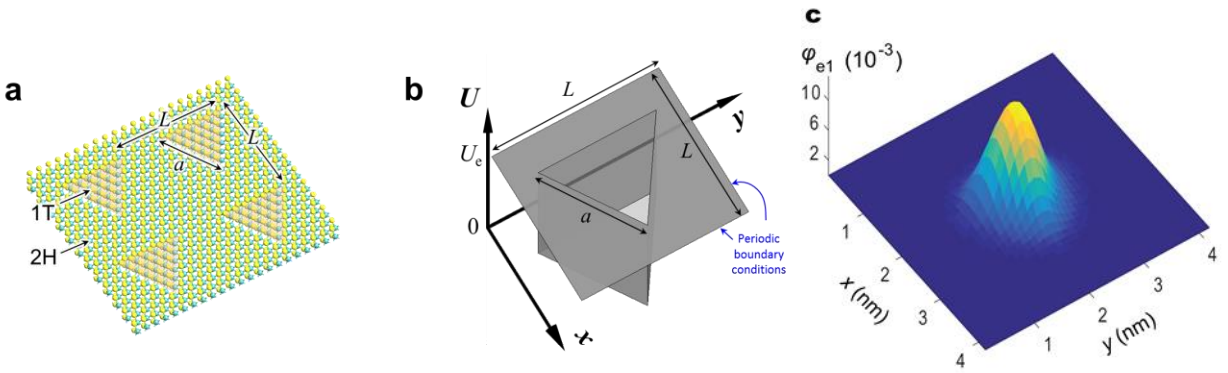

Figure S7. **(a)**, Schematic of the crystal structure of 1T-2H superlattice; **(b)**, Corresponding potential profile for electrons; **(c)**, Wave function of the first energy level Φ^{E1} at $\boldsymbol{k}$ = 0.

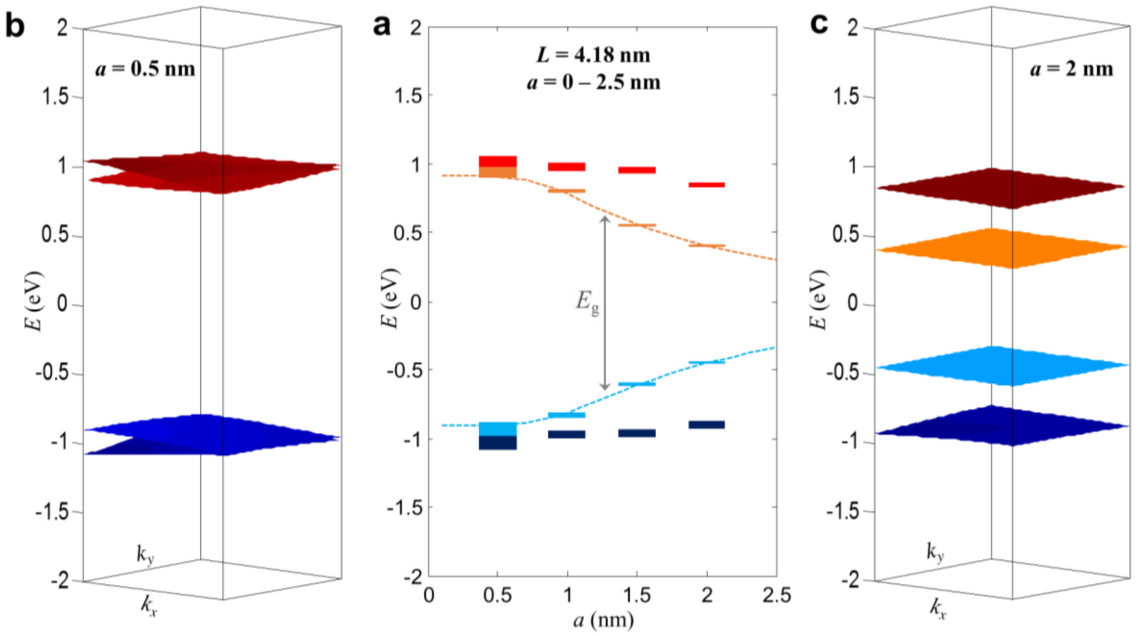

Figure S8. **(a)**, Band diagram vs. quantum well size *a* (triangle edge length) with L = 4.18 nm. Dashed lines: band edges vs *a*, for electrons and holes in MoS_2. **(b)**, Band structure of the superlattice with a = 0.5 nm; **(c)**, Band structure of the superlattice with a = 2 nm.

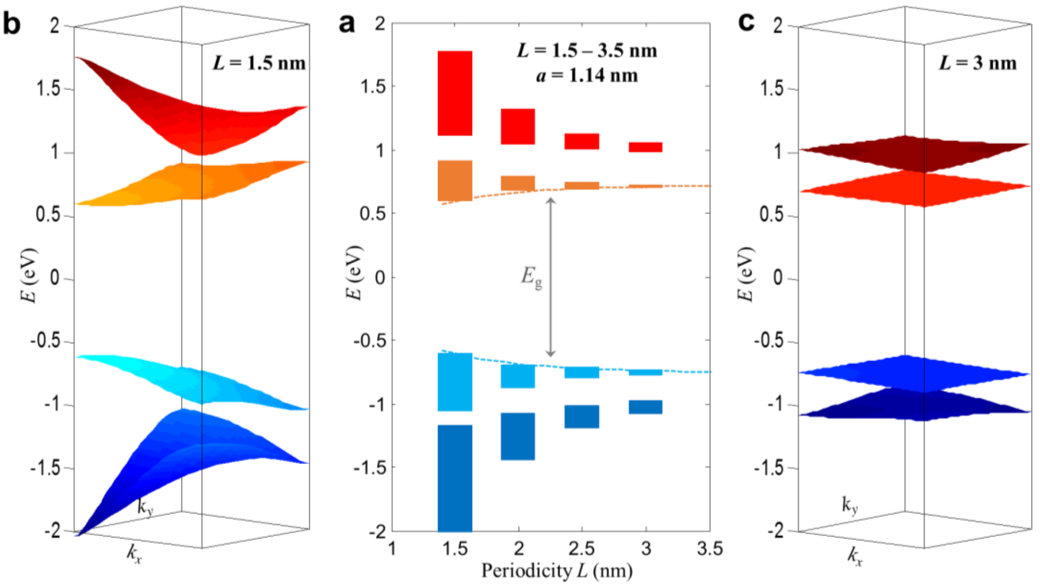

Figure S9. **(a)**, Band diagram vs. superlattice periodicity L. a = 1.14 nm. Dashed lines: band edges vs L, for electrons and holes in MoS_2. **(b)**, Band structure of the superlattice with L = 1.5 nm; **(c)**, Band structure of the superlattice with L = 3 nm.

S6. Original data

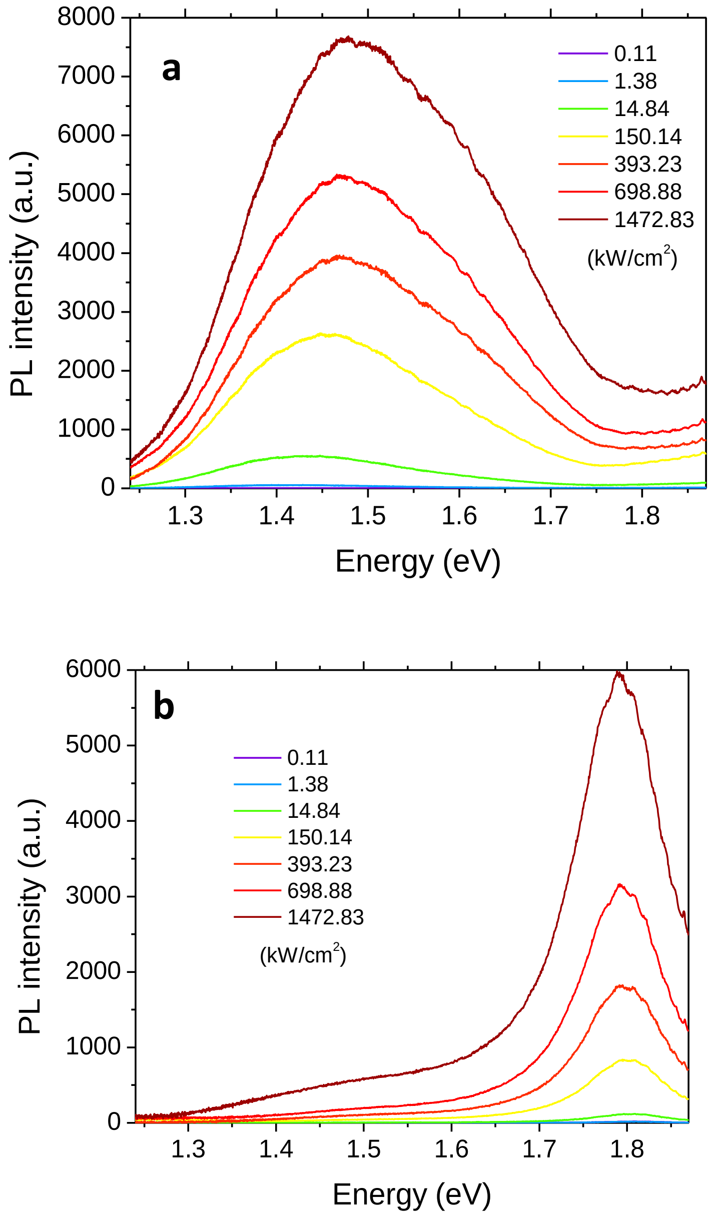

Figure S10. **(a)**, Original data for Fig 3a. **(b)**, Original data for Fig. 3b (in the main article).

References

1. Tongay, S. *et al.* Defects activated photoluminescence in two-dimensional semiconductors: interplay between bound, charged, and free excitons. *Sci. Rep.* **3,** 2657 (2013).

2. Chow, P. K. *et al.* Defect-induced photoluminescence in monolayer semiconducting transition metal dichalcogenides. *ACS Nano* **9,** 1520–1527 (2015).

3. Wang, Z., Ning, S., Fujita, T., Hirata, A. & Chen, M. Unveiling three-dimensional stacking sequences of 1T phase MoS_2 monolayers by electron diffraction. *ACS Nano* **10,** 10308–10316 (2016).

4. Lukowski, M. A. *et al.* Enhanced hydrogen evolution catalysis from chemically exfoliated metallic MoS2 nanosheets. *J. Am. Chem. Soc.* **135,** 10274–10277 (2013).

5. Perdew, J. P. & Zunger, A. Self-interaction correction to density-functional approximations for many-electron systems. *Phys. Rev. B* **23,** 5048–5079 (1981).

6. Rasmussen, F. A. & Thygesen, K. S. Computational 2D materials database: Electronic structure of transition-metal dichalcogenides and oxides. *J. Phys. Chem. C* **119,** 13169–13183 (2015).

7. Atomistix ToolKit version 13.8, QuantumWise A/S (www.quantumwise.com).

8. in *CRC Handbook on Chemistry and Physics* 12–114 (2008).
